# Holistic care and complex needs: unveiling the full potential of modern nursing

**DOI:** 10.3325/cmj.2024.65.530

**Published:** 2024-12

**Authors:** Ljerka Pavković, Dubravka Jakšetić, Silvija Marić, Dorja Vočanec, Aleksandar Džakula

**Affiliations:** 1 The Association of Nurses and Technicians of the Homeland War, Zagreb, Croatia; 2 Health Center Gospić, Gospić, Croatia; 3 County General Hospital Požega, Požega, Croatia; 4 Center for Health Systems, Policies and Diplomacy, Andrija Štampar School of Public Health, University of Zagreb School of Medicine, Zagreb, Croatia

## The challenge of focusing on holistic care

Fragmentation of care is undoubtedly one of the greatest challenges in modern health care. Modern medicine is strongly oriented toward detailed diagnostics and tailored interventions, delivering spectacular results daily. It is becoming increasingly precise, uncovering more details, and expanding its capacity to affect different health parameters. However, while focusing on details, it often misses the whole picture. The individuals with all their needs disappear, as do those who care for patients, along with the concept of holistic care. Holistic care has always been fundamental to the quality of health care. It is impossible to ensure adequate quality or patient well-being without considering the entire context in which care is provided. Modern health care heavily relies on resources, owing much of its success to advanced equipment and sophisticated knowledge. However, regardless of these achievements, the constant element remains the personal approach of professionals to patients, the ability to acknowledge each patient’s reality, and the capacity to respond appropriately. In this context, nurses hold a particularly important position among health care professionals (1). Their education, vocation, and role in the system are designed such that they must, and want to, respond to the specific needs of each patient and their environment. Nurses form a crucial link that ensures the continuity of care, reminding us of the fundamental values of humanity and empathy in everyday practice (2). Healthcare professionals carry a strong responsibility to help all individuals, regardless of their personal circumstances. Often, special effort is required to ensure that these values are not only accepted but consistently lived and implemented in practice. This is particularly challenging during times of war and crisis, when the principles of humanity, empathy, and an individualized approach face the greatest tests. Yet, it is precisely in such times that it becomes critical not only to preserve but to promote these values as the foundation of the health care profession and humanity. Even in the darkest moments, humanity can serve as a beacon, guiding us toward a better future.

## Addressing complex needs in extreme and everyday conditions

While extreme circumstances can unite people through shared dangers and risks, individual needs remain constant. This is why, during extraordinary times, it is essential to focus even more on individual needs. In other words, attention must be directed toward individuals with complex needs, as they are often the first to suffer due to their vulnerability and the last to receive care when it becomes available. In extreme situations, such individuals often top the list of the most vulnerable but end up at the bottom of the list of those receiving help and care (3,4). Therefore, it is critical to educate both professionals and laypeople to recognize and understand the complex needs of each individual. Early recognition of these needs, whether by laypeople or professionals, can significantly reduce the risk of severe consequences and enable quicker and more effective interventions. For this reason, it is essential to understand people around us even in normal circumstances, accept their specific needs as part of their identity, and be ready to act according to those specifics. This awareness and readiness are the foundation not only of prevention but also of ensuring better and timely care in all situations, whether during a crisis or in everyday life.

Many people, even in peacetime conditions, live in circumstances akin to wartime. Individuals, and entire populations living in rural and remote areas, often face planning and living in extreme conditions. Although this way of life can often offer the privilege of freedom and closeness to nature, when needs arise, these conditions suddenly become major challenges. Regardless of where and how we live, our complex needs exist and must be met. Moreover, individual complex needs become even more intricate when living in conditions without basic living necessities or with limited access to health care. In summary, complexity rarely comes alone; it is often compounded by additional barriers, creating challenges that require specific approaches and solutions. Such circumstances demand additional effort from the system and the community to ensure timely and adequate care for everyone.

## Complex patients as an opportunity to apply comprehensive nursing expertise

Nurses, thanks to their education, practical experience, and unique position in relation to patients, play a key role in caring for complex patients. Their competencies enable them to recognize not only the individual needs of patients but also the needs of caregivers, which is fundamental for realistic and timely detection of complex needs. Nurses have a unique and comprehensive perspective on the entire process of patient care. Unlike physicians, who often focus on specific aspects of medical care and spend limited time with patients, nurses are continuously involved in all stages of treatment. They monitor not only the clinical aspects but also the psychosocial needs of patients, providing support throughout all phases of recovery. Thanks to this comprehensive role, nurses naturally assume a coordinating function within the health care system. Their ability to see the entire process allows them to organize the patient’s journey through all the necessary care points, ensuring proper sequencing of interventions, optimal communication among teams, and a holistic approach that places the patient at the center of treatment. Such coordination not only increases efficiency but also significantly enhances the quality of health care (5,6). The knowledge and skills of nurses, gained through continuous patient care, enable them to provide holistic and comprehensive care. More importantly, their expertise allows them to design and implement care plans that are not limited to the specific conditions of health care institutions but are adapted to the everyday lives of patients, their families, and communities (7). This approach ensures quality and sustainable care, regardless of the circumstances or environment in which patients find themselves.

Developing programs for the care of complex patients or regulating health care to ensure comprehensive addressing of their needs does not represent new regulation of nursing work. On the contrary, addressing the issue of complex patients provides nurses with more space to act in a way they know best. This opportunity allows them to fully apply their professional knowledge and skills, fulfilling their role as professionals with a calling and mission that has accompanied their profession from its very beginnings. Regulating care for complex patients means assuming new tasks and responsibilities for many professions, while for nurses, it represents an opportunity to realize their full potential and capacity. This contributes not only to the well-being of patients and their caregivers but also strengthens and develops the entire nursing profession, positioning it as a key stakeholder in the health care system.
